# Improving model construction of profile HMMs for remote homology detection through structural alignment

**DOI:** 10.1186/1471-2105-8-435

**Published:** 2007-11-09

**Authors:** Juliana S Bernardes, Alberto MR Dávila, Vítor S Costa, Gerson Zaverucha

**Affiliations:** 1 COPPE, Programa de Engenharia de Sistemas e Computação, Universidade Federal do Rio de Janeiro, Rio de Janeiro, Brazil; 2 Instituto Oswaldo Cruz Fiocruz, Rio de Janeiro, Brazil; 3 DCC-FCUP e LIACC, Universidade do Porto, Porto, Portugal

## Abstract

**Background:**

Remote homology detection is a challenging problem in Bioinformatics. Arguably, profile Hidden Markov Models (pHMMs) are one of the most successful approaches in addressing this important problem. pHMM packages present a relatively small computational cost, and perform particularly well at recognizing remote homologies. This raises the question of whether structural alignments could impact the performance of pHMMs trained from proteins in the *Twilight Zone*, as structural alignments are often more accurate than sequence alignments at identifying motifs and functional residues. Next, we assess the impact of using structural alignments in pHMM performance.

**Results:**

We used the SCOP database to perform our experiments. Structural alignments were obtained using the 3DCOFFEE and MAMMOTH-mult tools; sequence alignments were obtained using CLUSTALW, TCOFFEE, MAFFT and PROBCONS. We performed leave-one-family-out cross-validation over super-families. Performance was evaluated through ROC curves and paired two tailed t-test.

**Conclusion:**

We observed that pHMMs derived from structural alignments performed significantly better than pHMMs derived from sequence alignment in low-identity regions, mainly below 20%. We believe this is because structural alignment tools are better at focusing on the important patterns that are more often conserved through evolution, resulting in higher quality pHMMs. On the other hand, sensitivity of these tools is still quite low for these low-identity regions. Our results suggest a number of possible directions for improvements in this area.

## Background

Hidden Markov models (HMMs) [1] are probabilistic models utilized in pattern recognition problems. HMMs were initially used for speech recognition tasks [2]. Nowadays, HMMs are being applied successfully to several molecular biology problems, including gene finding [3,4], multiple sequence alignment [5-7], protein structure prediction [8-10], and many others. One particularly important application of HMMs is in *remote homology detection *between protein sequences. Remote homology detection is the problem of finding homology between sequences, when the actual sequence identity is low (usually, lower than 30%). HMMs can be used by first training an HMM to represent a group of homologue sequences, and then matching a sequence against this HMM. The HMMs used to represents groups of homologues sequences are called *profile hidden Markov models (pHMMs) *[11,12]. Several studies have shown pHMMs to perform better than methods based on sequence similarity only [13,14], such as BLAST [15] and FASTA [16], and than methods based on position-specific scoring matrices (PSSMs) [17], such as PSI-BLAST [18].

A pHMM is therefore a probabilistic model built from a multiple alignment of related sequences. The two major programs that apply pHMM for remote homology detection are HMMER [19] and SAM [20]. Both programs are widely used within the Bioinformatics community. Namely, HMMER was used to build the PFAM database [21], and SAM was used to build Super-family [13]. In these tools, an alignment is represented by creating a sequence of nodes, usually one node per alignment column. Each node is composed of three states: *match (M), insert (I) *and *delete (D)*. Match states model conserved regions in the alignment. Insert and delete states model *indel *regions.

Profile HMMs have probabilities on two events: a transition from a state to another state, and the probability that a specific state will emit a specific character (say, a specific amino-acid when comparing proteins). Only match and insert states generate characters. Delete states are *quiet*. Therefore, each match and insert state has an *emission probability distribution*. In the case of proteins, the distribution will have 20 entries, one per amino acid.

Transitions define the structure of the pHMM. Systems such as SAM [20] allow transitions between all types of states, totaling 3 transitions per state, hence 9 per node. This is not always the case, the HMMER system relies on the Plan7 model [19], which disallows *I *→ *D *and *D *→ *I *transitions.

Performance of a pHMM critically depends on the quality of the estimated emission and transition probabilities. Emission probabilities are obtained by counting amino-acid frequencies at each match column. Unfortunately, the global alignment will usually have too few sequences to estimate all the parameters with sufficient confidence. Priors, such as mixtures of Dirichlets components [22], are used to compensate for the small sample size and avoid over-fitting. A second major issue when estimating parameters is the relationship between the sequences themselves. Clearly, the information that a residue is better conserved across a number of very different sequences should carry more weight than the information the residue is conserved across a large number of very similar sequences. Most pHMMs thus include a sequence weighting step, which may be based on sequence trees, as in HMMER [23], or in entropy, as in SAM [24]. In all cases, closer sequences carry less weight than more divergent sequences. Last, notice that the total weight of the sequences governs how much we trust the sequences versus the prior. Increasing the total weight of the sequence counts over the priors reinforces our trust in the sequence data, but may lead to over-fitting.

To the best of our knowledge, Madera and Gough were the first ones to systematically compare the performance of the two systems [25]. Their comparison studied the performance of the two tools over two protein families, globins and cupredoxins, using the nrdb90 database [26], and in an all-against-all experiment in the SCOP database [27]. Several alignment strategies were used, including: manual alignment on globins and cupredoxins, SAM-T99 [28] seeded from a single protein, WU-BLAST [29] search from the seed protein followed by CLUSTALW [30]. The authors show that the initial multiple alignment can significantly affect performance, and that the T99 package generates good quality multiple alignments. Their results further suggested that SAM had better model quality than HMMER. Wistrand and Sonnhammer [31] further evaluated the two systems. The experiments relied on SCOP for a high quality database of labeled hierarchies of protein domains. The authors explicitly avoided conditioning on the use of particular programs to perform the initial multiple alignment. Instead, they used the PFAM alignment database. The authors concluded that SAM's model estimation is superior, due to a better usage of priors, which avoids over-fitting. On the other hand, HMMER's model scoring is more accurate, probably due to a better null model.

Madera and Gough's work showed the importance of multiple alignment for HMMER performance. It has been observed that protein three-dimensional structures are remarkably stable with respect to amino acids sequences [32]. This suggests that alignments derived from structural information should identify motifs and functional residues accurately. In this direction, Jones and Bateman [33] assessed the performance of pHMMs derived of structural alignments versus sequence alignments. The benchmark was obtained from the PFAM and HOMSTRAD [34] databases. HOMSTRAD is a curated database of structure-based alignments for homologous protein families and PFAM is a large collection of multiple sequence alignments and hidden Markov models covering many common protein domains and families. To build up a mapping of HOMSTRAD and PFAM families, the sequences of each HOMSTRAD family were searched against PFAM using HMMER. Each HOMSTRAD family was thus made to correspond to a single PFAM *family*. Theses PFAM memberships were considered the true positive data set. To provide sequence alignments, the sequences of each HOMSTRAD family were realigned using CLUSTALW and TCOFFEE [35]. The authors concluded that the use of structure information to increase alignment accuracy does not aid homologue detection with pHMMs. However, their experiments considered sequences with different degrees of identity, from 20% up to the 80%, and the author did not applied his experiments to proteins in the *Twilight Zone*, where identity between amino-acids sequences is a weaker indicative of the evolutionary relationship.

This study investigates the contribution of using structural alignments to build pHMMs for remote homology detection. Therefore, our experiments consider proteins with identity below 30%. We performed our studies by analyzing the performance of these tools on SCOP *super-families*. Under these conditions, we show that pHMMs derived from structural alignments perform significantly better than pHMMs derived from sequence alignments. We show that accuracy alignment is not directly related to alignment identity. Although structural alignments often present smaller identity than sequence alignments, the best quality alignments based on structural information are generally considered to derive from structural superposition. We compare the performance of two HMMs packages, HMMER and SAM, when the two different kind of alignments were used. Our results show that HMMER based on structural alignment to outperform SAM for such remote homologues.

## Methods

We compare sequence-based and structure-based multiple alignment packages on the SCOP Protein Database. We evaluated experimentally the performance of the HMMER and SAM packages using alignments from four sequence and two structural multiple-alignment packages. All data sets and perl scripts used in this study are freely available from the web site [36].

### Multiple Alignment Tools

We used CLUSTALW [30], TCOFFEE [35], MAFFT [37], and PROBCONS [38] packages to provide sequence alignments based on primary structure. CLUSTALW is one of the most widely used tools for multiple sequence alignment. TCOFFEE has been reported to achieve significantly better quality alignments than CLUSTALW [39]. MAFFT is a series of five progressive alignment programs, we used L-INS-i, an algorithm based progressive alignment with iterative refinement. We used the 3DCOFFEE [40] and MAMMOTH-mult [41] packages to provide structural alignments. 3DCOFFEE extends TCOFFEE with structural alignment information. MAMMOTH-mult is a package that seems to achieve good performance by focusing on structural information.

CLUSTALW is a progressive alignment algorithm [42]. First, it derives a guide tree and then uses a greedy search over aligned clusters of sequences. Although, it perform faster and uses less memory than other programs, arguably it is less accurate or scalable than modern ones.

T-COFFEE also implements a progressive alignment algorithm. However, it tries to improve the quality of the initial pair-wise sequence alignment by considering the alignment between all the pairs as it executes every step in the progressive alignment algorithm. It presents high accuracy while sacrifices computation time and memory usage.

The MAFFT package includes five alignment programs. We used the recommended option, in this case, L-INS-i, that uses progressive aligner followed by iterative refinement.

PROBCONS uses a combination of probabilistic modeling and consistency-based alignment techniques. It introduces a novel scoring function, *probabilistic consistency*, based on paired hidden Markov models. Alignments are still performed progressively but a post-processing refinement step may apply.

The 3DCOFFEE aligner is based on TCOFFEE, but it uses pairwise structure comparison to improve accuracy. Pairwise structure comparison is performed by SAP if both structures are known [43]. If only one structure is known, 3DCOFFEE uses the Fugue threading method [44].

MAMMOTH is a progressive multiple alignment program that uses a sequence independent heuristic to obtain a fully structural alignment. It starts from a C*α *trace to obtain an alignment. Second, it finds an alignment of local structures based on computing a similarity score from the URMS metrics. Third, it finds similar local structures with their *Cα *close in Cartesian space.

### Profile-HMMs

We compare two arguably major profile Hidden Markov Model (pHMM) packages, HMMER and SAM.

The HMMER package was developed at the Sean Eddy's Lab, University of Washington Saint-Louis. It provides an open-source environment based on pHMMs for protein sequence analysis. Besides the PFAM database, HMMER is also at the heart of other databases, such as TIGRFAMs [45], and SMART [46]. In this work we used HMMER version 2.3.2, updated in 2003. HMMER requires at least two stages: model *building *and *scoring*. A third, recommended but optional stage, is model *calibration*: we have used it in this study.

In model building, HMMER distinguishes match alignment columns and insert alignment columns. HMMER assigns columns to match or insert states so as to maximize the posterior probability of the aligned sequences, given the model. By default, HMMER uses a Dirichlet mixture with 9 components for priors. Scoring was performed using the *Viterbi *algorithm. We used hmmbuild procedure to build HMMER models, and the hmmsearch for score. In our experiments we used HMMER default parameters.

The SAM package was developed at the University of California Santa Cruz; it is not open source but is free to academic use. One of the major SAM differences with respect to HMMER is the SAM-T2K script. This is an iterative procedure to generate multiple alignments and HMMs starting from a single sequence [28]. Moreover, the SAM team has worked on improving SAM through using information on structure protein [47], and prior probabilities [48]. SAM uses a standard profile HMM architecture with 9 transitions. Each alignment column correspond a node (match, insert and delete). In other words, SAM does not distinguish between match and insert columns. SAM uses a Dirichlet mixture with 20 components for priors and by default scores using the *forward *algorithm. We used modelfromalign to build the models and hmmscore to compute. In our experiments we used SAM default parameters.

### Experimental Methodology

Our experiments require structure coordinates for protein sets with low sequence identity. Therefore we used the SCOP database [27], version 1.67 with 6600 proteins sequences. SCOP is a manually inspected database of protein folds, and is particularly interesting for our study because it describes structural and evolutionary relationships between proteins, including all entries in the Protein Data Bank [49]. SCOP is thus an excellent data-set for evaluating the performance of remote homology detection methods, and it has been widely used for that purpose [31,50-53]. SCOP classifies all protein domains of known structure into a hierarchy with four levels: class, fold, super family, and family. In our study, we work at the super family level, which groups families such that a common evolutionary origin is not obvious from sequence identity, but probable from an analysis of structure and from functional features. We believe that this level best represents remote homologies.

Throughout, we used cross-validation [54] to compare the different approaches. First, we divided SCOP database by super family level. Next, from ASTRAL PDB40, we choose those super families containing at least two families and at least 20 sequences. We eventually tested 39 super families, as listed in Table 1. This whittled down the number of sequences we use for model building to 1137. Third, we implemented leave-one-family-out cross-validation. For any super family *x *having *n *families, we built *n *profiles, such that each profile *P *was built from the sequences in the remaining *n *- 1 families. Thus, the *n *- 1 sequences form the training set for profile *P*. The test set for profile *P *will be the remaining sequences (test positives) plus all other database sequences (test negatives).

**Table 1:** Super-family SCOP-Ids

a.1.1.	a.138.1.	a.25.1.	a.26.1.	a.3.1.	a.39.1.	a.4.1.	b.121.4.
b.18.1.	b.29.1.	b.36.1.	b.47.1.	b.55.1.	b.60.1.	b.6.1.	b.71.1.
b.82.1.	c.1.10.	c.23.1.	c.26.1.	c.36.1.	c.52.1.	c.55.1.	c.55.3.
c.67.1.	d.108.1.	d.14.1.	d.144.1.	d.15.1.	d.153.1.	d.169.1.	d.3.1.
d.58.7.	d.92.1.	g.3.11.	g.3.6.	g.3.7.	g.37.1.	g.39.1.	

SCOP Super-families used in our experiments. We only consider super-families with at least 20 proteins and two or more families.

Note that in our experiments, none of the sequences in a test set had >30% sequence identity with any protein in the corresponding training set. Results were graphically analyzed by building ROC. We experimented with e-values between 10^-50 ^and 10 to obtain the curves. Finally, we have used the paired two tailed t-test to assess significance. We consider a result with *p *≤ 0.02 (i.e. 98% of confidence) to be significant.

## Results

### Alignment Profile

As a first step, we categorize our alignment data set according to both the number of sequences, and the average length of sequences within SCOP super-family. In our data set, the number of sequences per super-family ranges from 3 sequences in the smallest super-family up to 44 sequences for the largest super-families. In average, we worked with 23 sequences per alignment. Comparing with previous work on aligning families [33], we observe that super-families give us much more training examples to construct the pHMMs. Regarding sequence length, the average sequence length within SCOP super-families is well distributed in the interval between small sequences with less than 50 residues to large sequences with up to 400 residues. In average, we worked with sequences of 193 residues.

Next, we discuss alignment *profile *as measured by sequence identity, by alignment length and by percentage of gaps. Table 2 shows the average gap percentage and average alignment length in the alignments obtained from the six alignment tools in our study: CLUSTALW, TCOFFEE, MAFFT, PROBCONS, 3DCOFFEE, and MAMMOTH.

**Table 2:** Alignment Length and Gap Percentage and by Alignment Tool

	Length (residues)	Gap%
CLUSTALW	318	41
TCOFFEE	474	59
MAFFT	382	51
PROBCONS	540	59
3DCOFFEE	495	60
MAMMOTH	413	53

First, we assess average alignment length. CLUSTALW seems to generate the smallest alignments, with in average 318 residues. MAFFT and MAMMOTH generate longer alignments, in average around 400 residues. The longest alignments are generated by PROBCONS followed by COFFEE family. Notice that 3DCOFFEE generates somewhat longer alignments than TCOFFE. Next, we measure the percentage of gaps within alignments. CLUSTALW introduced the smallest gap percentage. MAFFT produced alignment with less gaps than MAMMOTH. The COFFEE family and PROBCONS present the longest alignments and have the highest percentage of gaps.

**Figure 1:**
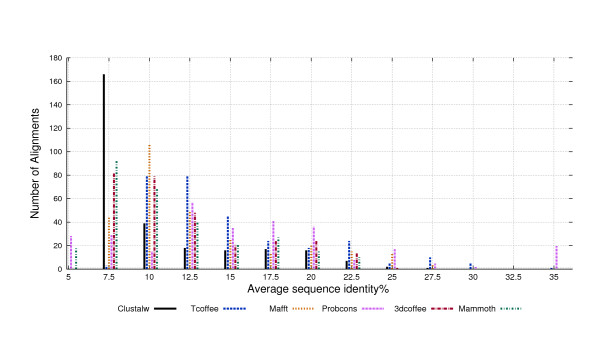
**Multiple Alignments Identity**. Average identity across the different alignment tools.

Next, we compared sequence identity within alignments. The Figure 1 shows average identity by alignment for the six alignment tools in our study. The figure shows that sequence identity is low as expected, most often below 30%. In fact, average identity as recognized by PROBCONS is of 16.76%, with 15.4% for TCOFFEE, 13.81% for MAFFT, 13.01% for 3DCOFFEE, MAMMOTH with 12%, and only 10.9% for CLUSTALW. Figure 1 does show that the PROBCONS tool generates alignments with "most identity". Indeed, it can recognize a number of alignments with more than 30% identity. PROBCONS also seems to produce alignments well distributed into all identity ranges. TCOFFEE performs almost as well as PROBCONS. Most MAFFT alignments are below 12% identity, but it can recognize alignments with up to 27.5% of identity. On the other hand, CLUSTALW seems to perform badly on a large number of cases. Most CLUSTALW alignments recognize between 7.5% and 10% identity and CLUSTAW further shows the lowest identity average. As regards the structural alignment tools 3DCOFFEE recognizes more identity than MAMMOTH, but the less than the related tool, TCOFFEE. MAMMOTH recognizes less identity than 3DCOFFE.

Gap introduction is clearly related with alignment length, and thus with identity. In general, sequence alignment tools need to introduce gaps to preserve identity across sequences. As a case in point, PROBCONS achieves the highest average identity, but 60% of PROBCONS alignments were gaps. We observed a similar pattern in TCOFFEE alignments. MAFFT achieves less identity but also introduces few gaps. Analogously, CLUSTALW presented the lowest identity average, and also introduced the smallest number of gaps in its alignments. Comparing the structural alignments, 3DCOFFEE achieved higher average identity than MAMMOTH, and also introduces more gaps than MAMMOTH. A Pearson test shows the correlation between alignment percentage and gaps to be indeed quite high, at 94%.

### HMMER Performance

We assessed HMMER performance using multiple alignments generated by CLUSTALW, TCOFFEE, MAFFT, PROBCONS, 3DCOFFEE, and MAMMOTH. For a super-family with *N *elements, the results indicate whether models trained on *N *- 1 families can predict the sequences in the remaining family. Please see the Methods section above for further discussion on the experimental methodology.

**Figure 2:**
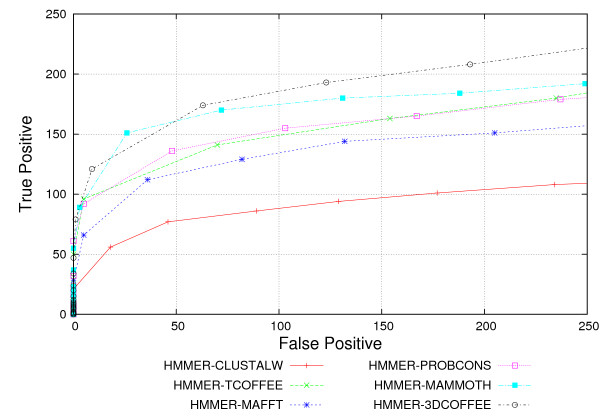
HMMER ROC Curves. HMMER performance for all alignment tools, as measured by ROC curves.

Figure 2 shows the ROC curves [55] for the whole database. Table 3 further shows t-test results, where we compare aligners against all the others. We observed best results with 3DCOFFEE-generated alignments, but HMMER-MAMMOTH also performed well. According to a paired two tailed t-test [54] over the set of experiments the results from theses tools are significantly better than for the other tools, although the difference between HMMER-MAMMOTH and HMMER-3DCOFFEE is not statistically significant. The results also show HMMER-MAFFT, HMMER-TCOFFEE, and HMMER-PROBCONS performing similarly. Our results do not show significant differences in the performance of the models generated by these tools. On the other hand, one should remark that HMMER-3DCOFFEE significantly outperform HMMER-TCOFFEE. Last, HMMER-CLUSTALW performs much worse than the other tools. Again, a t-test showed this results to be statistically significant.

**Table 3:** HMMER Significance Results

	3DCOFFEE	MAMMOTH	PROBCONS	MAFFT	TCOFFEE
CLUSTALW	**10**^**-8**^	**10**^**-8**^	0.015	0.61	**0**. **01**
TCOFFEE	**10**^**-6**^	**10**^**-6**^	0.12	0.02	
MAFFT	**10**^**-7**^	**10**^**-7**^	0.02		
PROBCONS	**10**^**-6**^	**10**^**-6**^			
MAMMOTH	0.54				

Paired two-tailed test results when comparing performance of HMMER alignments for all the multiple alignment tools.

For better understanding, we further partition our results according to identity ranges. Given that our best results were obtained from HMMER-3DCOFFEE, we rely on 3DCOFFEE as our measure of sequence identity.

**Figure 3:**
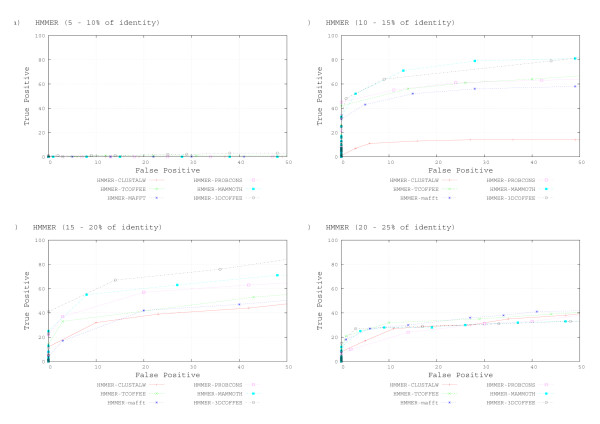
**HMMER ROC Curves by identity range**. HMMER Performance for all Alignment Tools, as Measured by ROC Curves. ROC curves were produced interposed in 5% sequence identity intervals. A – identity bellow of 10%. B – identity between 10% and 15%. C – identity between 15% and 20%. D – identity between 20% and 25%. E – identity above of 25%.

Figures 3-a to 3-d show ROC curves for sequences interposed in 5% sequence identity intervals (notice that 3DCOFFEE could not find more than 25% identity throughout). All tools do very badly when identity is below 10%. HMMER-3DCOFEE and HMMER-MAMMOTH dominate the other tools on the range 10–15%. Sequence based aligners perform similarly, except for CLUSTALW: models trained with HMMER-CLUSTALW alignment have very low sensitivity. On the range 15–20% HMMER-3DCOFFEE alignments perform clearly better than the other tools. The difference is less clear for HMMER-MAMMOTH, especially for low specificity. Notice the clear difference between HMMER-3DCOFFEE and HMMER-TCOFFEE at this range. Models trained from CLUSTALW still have lower sensitivity, but the gap is less clear. Last, above 20% the difference between tools is not very clear, tools tend to have similar recalls as we lower specificity. Notice that both HMMER-COFFEE tools now perform quite similarly and that HMMER-CLUSTALW also achieves results similar to the other tools.

### SAM Performance

**Figure 4:**
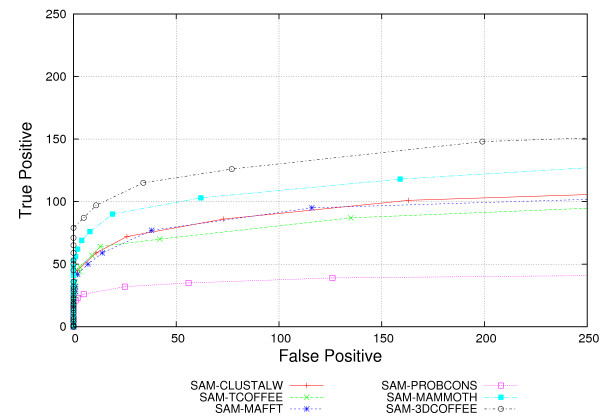
SAM ROC Curves. SAM performance for all alignment tools, as measured by ROC curves.

We assessed SAM performance using default parameters. We use the ROC curves in Figure 4 to show overall performance. Table 4 further shows t-test results. A first observation is that SAM recognizes much less true positives than HMMER. SAM recognizes around 100 true positives for 50 false positives, whereas HMMER recognizes more than 150 true positives at the same number of false positives.

**Table 4:** SAM Significance Results

	3DCOFFEE	MAMMOTH	PROBCONS	MAFFT	TCOFFEE
CLUSTALW	**10**^**-8**^	**10**^**-8**^	**10**^**-3**^	0.02	**10**^**-4**^
TCOFFEE	**10**^**-6**^	**10**^**-6**^	0.23	0.08	
MAFFT	**10**^**-6**^	**10**^**-6**^	0.03		
PROBCONS	**10**^**-5**^	**10**^**-5**^			
MAMMOTH	0.28				

Paired two-tailed results when comparing performance of SAM alignments for all the multiple alignment tools.

Best results were achieved with SAM-3DCOFFEE, followed by SAM-MAMMOTH. Difference between the two was not statistically significant. The pHMMs derived from sequence alignments achieved worse results, but surprisingly SAM-CLUSTALW and SAM-MAFFT actually operate significantly better than SAM-PROBCONS. The difference between SAM-TCOFFEE and SAM-PROBCONS is not significant. On the other hand, there is a clear difference between SAM-CLUSTALW, SAM-PROBCONS and SAM-TCOFFEE.

**Figure 5:**
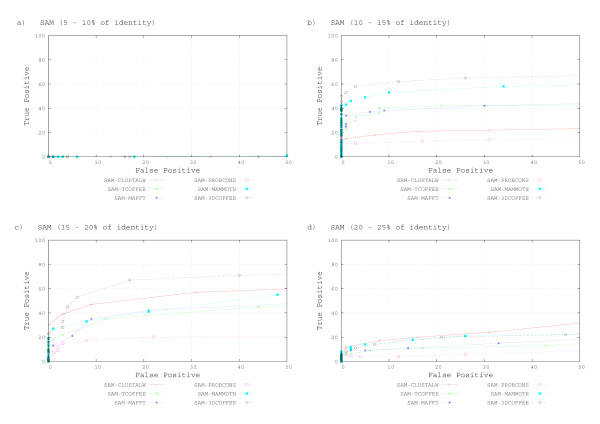
SAM ROC Curves by Identity Range. SAM performance for all alignment tools, as measured by ROC curves. ROC curves were produced interposed in 5% sequence identity intervals. A – identity bellow of 10%. B – identity between 10% and 15%. C – identity between 15% and 20%. D – identity between 20% and 25%. E – identity above of 25%.

Figures 5-a to 5-d show ROC curves for sequences interposed in 5% sequence identity intervals. The constructed models are simply not sensitive under 10% identity. The range 10–15% shows clear superiority of the structural aligners SAM-3DCOFFEE and SAM-MAMMOTH. SAM-CLUSTALW and SAM-PROBCONS do badly: SAM-PROBCONS has particularly low specificity. The picture changes for 15–20% identity: SAM-3DCOFFEE still does well. Notice that SAM-CLUSTALW actually does quite well at this range, but that SAM-PROBCONS performs badly. SAM-MAMMOTH, SAM-MAFT and SAM-TCOFFEE perform similarly. Last, we observe that most alignments have similar performance at above 20% identity. There is still some difference between SAM-TCOFFEE and SAM-3DCOFFEE. As for other identity ranges, SAM-PROBCONS performs badly.

### HMMER and SAM Performance

**Figure 6:**
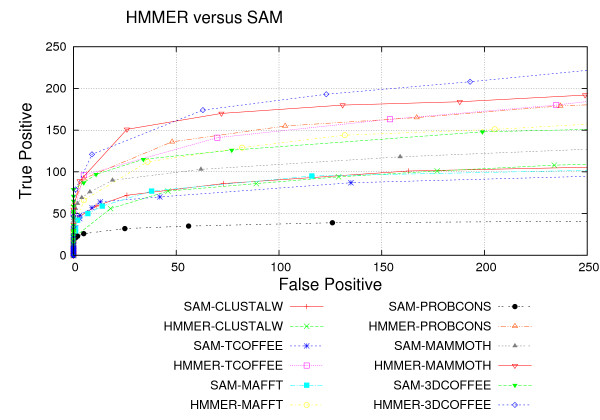
**HMMER-SAM ROC Curves**. HMMER and SAM performance for all alignment tools, as measured by ROC curves.

Last, we compare the overall performance of the HMMER and SAM packages using the different alignment tools. Figure 6 shows that best overall results were obtained by HMMER using the structural alignments derived from MAMMOTH and 3DCOFFEE. These are the only cases where the tools could recognize more than 200 sequences. The difference from HMMER-MAMMOTH or HMMER-3DCOFFEE to SAM-MAMMOTH or SAM-3DCOFFEE is statistically significant, the Table 5 shows t-test results. Regarding the pHMMs derived from sequence alignment, HMMER-TCOFFEE performed better than SAM-TCOFFE. The exception are the alignments generated by CLUSTALW: SAM-CLUSTALW achieves better results than HMMER-CLUSTALW. All results involving HMMER and SAM pHMM performances are statistically significant.

**Table 5:** HMMER-SAM Significance Results

HMMER-CLUSTALW × SAM-CLUSTALW	0.01
HMMER-TCOFFEE × SAM-TCOFFEE	**10**^**-4**^
HMMER-MAFFT × SAM-MAFFT	**10**^**-6**^
HMMER-PROBCONS × SAM-PROBCONS	**10**^**-6**^
HMMER-3DCOFFEE × SAM-3DCOFFEE	**10**^**-5**^
HMMER-MAMMOTH × SAM-MAMMOTH	**10**^**-5**^

Paired two tailed results when comparing performance of HMMER and SAM for the all the multiple alignment tools.

**Figure 7:**
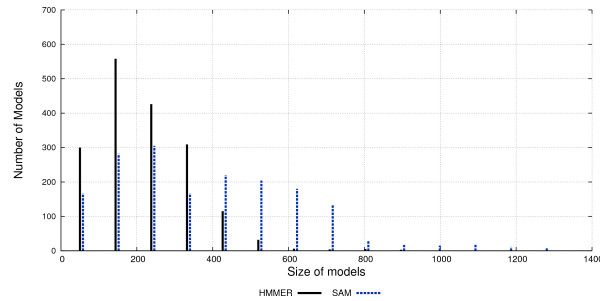
Models Sizes . Distribution of models sizes for pHMMs derives from HMMER and SAM.

In order to better understand the difference between models, Figure 7 shows how the distribution of models sizes for the pHMMs generated by HMMER and SAM. There is a very clear difference in model size with HMMER generating much shorter alignments.

## Discussion

Detecting remote homologue is an important, but hard, problem, as there is high divergence between training sequences. Several approaches have been proposed to improve pHMMs performance in these conditions [56-58]. A natural approach is to use protein structural information to improve model quality [52,59,60]. In this work, we investigated whether one can leverage preexisting tools, such as SAM and HMMER, by applying multiple alignments based on structural information.

The major question we address is therefore whether pHMMs for remote homology detection will benefit from structure alignments. Previous work showed negative results [33] on sequences having identity between 20–80%. To study whether similar results would apply to the *Twilight Zone*, we performed experiments comparing performance across SCOP super-families. We used the SCOP database, as this is the standard database with structural information being used in most related studies. Throughout, we used leave one-family out cross-validation instead of leave one-sequence out, as we believe this most closely represents the problem of finding a novel remote homologue.

Our focus was on how HMMER and SAM can benefit from structural information. We therefore used the two tools with *external *alignments. SAM is often used together with the T-99 aligner (that can use secondary but not tertiary information).

Our results show clear benefit from using structural aligners. The benefit was noticeable for both SAM and HMMER. A detailed analysis shows that the improvement was obtained in the 10–20% identity range in both cases. Below 10% identity is too low, and the tools do not generate useful models. Above 20% identity, both for SAM and for HMMER alignments from the sequence based tools start performing comparably to the structural aligners, a results consistent with the literature.

Studying the difference between TCOFFEE and 3DCOFFEE is particularly enlightening, as the two aligners mostly differ on the use of structural information. There is indeed a significant difference between the two tools in this study, and the difference applies both to SAM and HMMER models. Moreover, the difference stems from lower identity, in the 10–20% identity range, and disappears as sequences become more conserved.

We found no correlation between alignment size and model performance. PROBCONS consistently generates the longest alignments, but it does not outperform the other tools. MAMMOTH tends to generate relatively short alignments, and performs well in this study. This would suggest that the problem is not just finding conserved regions, but that the aligners might be reporting regions to be conserved when they are not.

Although our key results are similar for SAM and HMMER, we did observe a number of interesting differences. First, our studies indicate better sensitivity of HMMER-based models than of SAM based models. Second, some aligners perform quite differently when they are used by SAM and by HMMER. Namely, PROBCONS generated alignments performs particularly badly with SAM. In fact, SAM-CLUSTALW actually outperforms SAM-PROBCONS.

We believe that the explanation for both phenomena lies in the way that HMMER and SAM treat their input alignments. SAM is designed to be used together with the T99 aligner, and thus each column in the multiple alignment results in a state on the resulting pHMM. In contrast, HMMER is designed to be used with external aligners. Thus, it implements a MAP algorithm to estimate the actual number of states. Our results do show this MAP algorithm to significantly reduce the number of states for HMMER.

## Conclusion

Finding remote homologue is a hard, but important problem in molecular biology. We study the performance of two pHMM based tools, SAM and HMMER, when provided with structural and sequential alignments. We reach two main conclusions. First, structural alignments are very important in low-identity regions, below 20%. Using structural information can significantly improve performance in this task. On the other hand, our results indicate that alignments are low quality, even in the best case. Thus sensitivity is still quite low: we achieved at most 200 of about 1000 sequences in our study.

We believe that there is still much open work in achieving best performance in recognizing remotely related proteins. Our results suggest a number of possible directions for improvements in this area. The good results obtained by 3DCOFFEE, which performs quite well both when compared to a tool such as MAMMOTH-mult, designed from the beginning to perform structural alignments, and when compared with the corresponding sequential aligner, TCOFFEE, suggests that similar improvements could be considered for other sequence aligners. Our results also show that structural identity does provide a good prior on alignment quality. In current approaches, this prior is only used to generate the alignments. It would be interesting to go one step further and to integrate this information with the model construction process itself.

## Authors' contributions

JSB carried out the studies. AMRD, GZ and VSC participated in the design and coordination of the study, and in the writing of the manuscript. All authors read and approved the final manuscript.
